# Books and Authors

**Published:** 1908-05

**Authors:** 


### BOOKS AND AUTHORS.

---

**Surgery:** Its Principles and Practice. In five volumes. By 66 eminent surgeons. Edited by **W. W. Keen,** M.D., Emeritus Professor of the Principles of Surgery and of Clinical Surgery, Jefferson Medical College, Philadelphia. Volume III. Octavo of 1132 pages, with 562 text-illustrations and 10 colored plates. Philadelphia and London: W. B. Saunders Company. 1908. (Per volume: cloth, $7.00; half morocco, $8.00, net prices.)

This volume deals with surgery of the head and neck, (Cushing & Andrews), of diseases of the thyroid gland (Kocher), the nose and its accessory sinuses (Smith), surgery of the larynx and trachea and of the thorax (Brewer), of the breast (Finney), of the mouth, teeth, and jaws (Owen), of the tongue (Da Costa), technic of abdominal surgery, surgery of the abdominal wall, and surgery of the peritoneum and retroperitoneal space (Munro), surgery of the esophagus (Gottstein), of the stomach (Robson), of the liver, gall-bladder and the biliary ducts (William J. and C. H. Mayo), surgery of the pancreas and spleen (Moynihan).

A most instructive section which is also of deepest interest comes first in the book, dealing with the surgery of the head. Time was when surgery was invoked only for traumatism of the head, but now operations are made even more frequently for disease of the brain or its meninges. Osteoplastic crainotomy is fully described, and the illustrations are superb. The surgery of the thorax and of the breast are two other chapters of great practical importance. Brewer and Finney have dealt with these topics in a scientifically complete manner, and have given beautifully accurate illustrations of these important procedures.

This entire volume is full of good things and is a fit companion to its predecessors. It reflects credit on American surgeons and American surgery, as well as to those foreigners who have made contributions to the book.

---

**Progressive Medicine,** Vol. X, March, 1908. A Quarterly Digest of Advances, Discoveries and Improvements in the Medical and Surgical Sciences. Edited by **Hobart Amory Hare,** M.D., Professor of Therapeutics and Materia Medica in the Jefferson Medical College of Philadelphia. Octavo, 284 pages. Lea Brothers & Co., Publishers, Philadelphia and New York. (Per annum in four cloth-bound volumes, $9.00; in paper binding, $6.00, carriage paid to any address.)

The surgery of the head, neck, and thorax forms the subject of the first section in this number which is prepared by Charles H. Frazier, and in which he deals with recent advances of regions covering nearly one half the body. It is most interestingly written and thoroughly detailed. The section on infectious diseases, including acute rheumatism and croupous pneumonia, is prepared by Robert B. Preble, and the selections have been discreetly made. Floyd M. Crandall takes up the diseases of children, and handles the topic with judgment. Infant foods and infant feeding, always of importance, are set forth in Dr. Crandall's article with scientific import. In the section on laryngology and rhinology. D. Braden Kyle gives the latest information on these topics, as does Arthur B. Duel on otology. These several articles set forth the practical advances in the topics with which they deal, written in an attractive manner, making them of great value to the general practitioner. The present number is one of the best of the recent issues of this periodical.

---

**A Textbook of Minor Surgery.** By **Edward Milton Foote,** A.M., M.D., Instructor in Surgery in the College of Physicians and Surgeons (Columbia University) New York. Octavo, pp. 778. Illustrated by 407 engravings from original drawings and photographs. New York and London: D. Appleton and Company. 1908. (Cloth, $5.00.)

Whoever undertakes a study of this book will be impressed early with its completeness. Without doubt it is the most comprehensive treatise on minor surgery published in America. It is indeed, a textbook, whereas all previous works on minor surgery have been mere handbooks. The large number of the illustrations, nearly all photographs of actual patients, bespeak a thoroughness with which the author has dealt with his topics. In this relation we may well quote the dedication, which is unusual in form and original in subject-matter. "This book is dedicated to the man at the point of the knife, for his grit and patience, and especially for his willingness to be photographed that others may profit by his misfortune." Surely every sick or injured person who submits himself to the camera for the benefit of his fellowmen is a philanthropist.

This author has taken up many topics for consideration not usually dealt with in a work on minor surgery, but which in our view belong here rather than in a textbook on general surgery. The section on minor surgical technic merits careful attention from every student and junior practitioner. The chapter on the roller bandage is the most complete dissertation on the subject extant, and never before has it been so profusely or completely illustrated. The chapter on surgical dressings, too, deserves patient attention. These are topics the novitiate must learn at once, and this work will afford the precise information needed. The book is handsomely printed on finely calendered paper and is a credit to all concerned in its making. Scientifically speaking, we regard it as the best surgical production of the year.

